# Graphene Oxide–Based Nanomaterials: An Insight into Retinal Prosthesis

**DOI:** 10.3390/ijms21082957

**Published:** 2020-04-22

**Authors:** Jia-Wei Yang, Zih-Yu Yu, Sheng-Jen Cheng, Johnson H. Y. Chung, Xiao Liu, Chung-Yu Wu, Shien-Fong Lin, Guan-Yu Chen

**Affiliations:** 1 Department of Electrical and Computer Engineering, College of Electrical and Computer Engineering, National Chiao Tung University, Hsinchu 300, Taiwan; jiawei@nctu.edu.tw (J.-W.Y.); shengjen@nctu.edu.tw (S.-J.C.); linsf5402@nctu.edu.tw (S.-F.L.); 2 Institute of Biomedical Engineering, College of Electrical and Computer Engineering, National Chiao Tung University, Hsinchu 300, Taiwan; zyyu.cm07g@nctu.edu.tw; 3 ARC Centre of Excellence for Electromaterials Science, Intelligent Polymer Research Institute, University of Wollongong, Wollongong, NSW 2500, Australia; johnsonc@uow.edu.au (J.H.Y.C.); xiaol@uow.edu.au (X.L.); 4 Department of Electrical Engineering, National Chiao Tung University, Hsinchu, 300, Taiwan; peterwu@mail.nctu.edu.tw; 5 Department of Biological Science and Technology, National Chiao Tung University, Hsinchu 300, Taiwan

**Keywords:** retinal prosthesis, electrodes, nanotechnology, graphene oxide, interface

## Abstract

Retinal prosthesis has recently emerged as a treatment strategy for retinopathies, providing excellent assistance in the treatment of age-related macular degeneration (AMD) and retinitis pigmentosa. The potential application of graphene oxide (GO), a highly biocompatible nanomaterial with superior physicochemical properties, in the fabrication of electrodes for retinal prosthesis, is reviewed in this article. This review integrates insights from biological medicine and nanotechnology, with electronic and electrical engineering technological breakthroughs, and aims to highlight innovative objectives in developing biomedical applications of retinal prosthesis.

## 1. Age-Related Macular Degeneration (AMD) and Common Treatment Strategies

**Figure 1:**
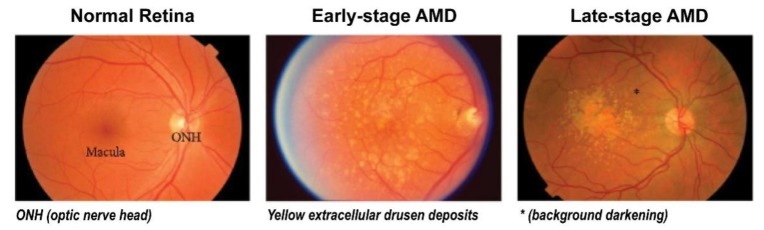
Clinical stages of age-related macular degeneration (AMD). Early-stage AMD shows yellow extracellular drusen deposits surrounding macular area. Late-stage AMD shows hypopigmentation or background darkening (*) around drusen, and a large number of drusen deposits are observed accumulated in the macular area. Adapted with permission from Jiangyuan et al. [15].

Retinal damage may lead to permanent loss of visual function and is considered to be an irreversible eye disease. In particular, age-related macular degeneration (AMD) is a disease that causes gradual degeneration of the central retina, and it is the leading cause of blindness in the elderly [1,2,3]. At present, macular degeneration is treated using different approaches, depending on location, size, and clinical symptoms of the lesion, with the treatments generally classified into early-, mid-, and late-stage approaches (Figure 1). Early-stage macular degeneration is treated with oral administration of antioxidants (e.g., vitamin C or lutein), but this treatment has limited efficacy [4,5]. Mid-stage AMD treatment consists of subjecting affected tissues to localized laser photocoagulation therapy, i.e., a laser beam is focused on peripheral tissues of the retina, to induce high-temperature burning [6,7]. This destroys neovascular vessels, thereby preventing leakage of vascular tissues and causing neovascular vessels in the lesion to shrink. However, this approach may cause damage to the adjacent normal tissues and can only be applied in positions far away from foveal lesions [8]. Therefore, although it prevents disease progression, it is unable to completely restore vision. Another approach is to employ photodynamic therapy (PDT). In PDT, an intravenously injected photosensitizer reaches the eye through blood circulation, where it gets concentrated in the wall of abnormal blood vessels, by binding to endothelial cells of the wall. Next, the cell-bound photosensitizer is irradiated with 690 nm red light, which penetrates the retinal tissues and reaches the hemorrhage layer, triggering photochemical reactions that produce single oxygen atoms and free radicals [9,10]. These cause oxidative damage to diseased cells and thereby destroy cellular function. This approach is selective, as it allows selective occlusion and closure of neovascular vessels while not destroying adjacent retinal tissues. In addition, this treatment approach does not produce high-temperature burning and has low damage to normal retinal tissues, making it amenable to applications in tissues close to foveal lesions. On the other hand, late-stage treatment includes injection of anti-vascular endothelial growth factor agents to suppress vascular endothelial growth factor (VEGF), which is a signal protein produced by retinal pigmented cells that stimulates the formation of blood vessels, and referred to as anti-VEGF therapy [11,12]. This represents a novel therapeutic strategy that has been proven to stabilize and improve vision of patients in a clinical setting and is, therefore, gradually replacing traditional laser photocoagulation therapy and photodynamic therapy (PDT). The agents directly injected into the vitreous cavity around the patient’s eyeball reduce the concentration of intraocular VEGF, inhibit vascular leakage and edema, and can retard neovascularization of the lesion, thereby preventing deterioration in vision. Moreover, this approach does not damage normal tissue structures, unlike laser photocoagulation therapy. However, this late-stage approach requires long-term medical therapy (lasting for at least one year), which may pose risks of developing complications such as endophthalmitis (bacterial infection), elevated intraocular pressure, cataracts, and retinal detachment, so it is necessary to conduct follow-up medical examinations at regular intervals [13,14].

For patients with late-stage AMD who cannot be treated with medication, the alternative is retinal transplantation or implantation of retinal prostheses [16,17]. At present, implantable electronic systems combined with cell-transplantation approaches can effectively enhance the repair efficacy of tissue and mitigate immunological rejection [18,19]. Moreover, a large number of research teams are working on the development of retinal prosthesis chip systems [20,21,22,23,24,25]. This involves replacement of damaged photoreceptor cells by retinal prosthesis with similar functions. The microelectrodes in the chips generate currents to stimulate retinal ganglion cells (RGCs), with the impulse signals transmitted back to the brain [26,27]. The technology of retinal prosthesis has developed gradually, allowing chip implants to electrically stimulate normal neurons to transmit signals. However, many problems still exist, including problems with the design of optical wireless transmission channels, signal resolution processing, and solar cell miniaturization [28,29,30,31]. On the other hand, there is a lack of research on the self-repair and regeneration of RGCs on these chips under electrical stimulation [32]. This is predominantly attributed to the lack of sufficient knowledge about the safety and stability of electrostimulation electrodes, as the electrical stimulation may induce cell apoptosis [33,34,35]. If the abovementioned challenges can be studied more in detail, retinal prosthesis will have even greater potential to serve as a novel strategy to restore vision.

## 2. Application of Retinal Prosthesis

About 40 years ago, several research groups proposed various electronic devices to convert visual information into hearing or tactile signals targeted toward blindness, such as sensory substitution devices [36]. In addition, Brindley et al. have implanted electrodes in the right occipital bone and integrated them to a radio receiver array, thereby stimulating the visual cortex or optic nerve, to restore vision [37]. However, many technical aspects of these methods remain challenging, such as device miniaturization, control of electrical stimulation, and power supply.

**Figure 2:**
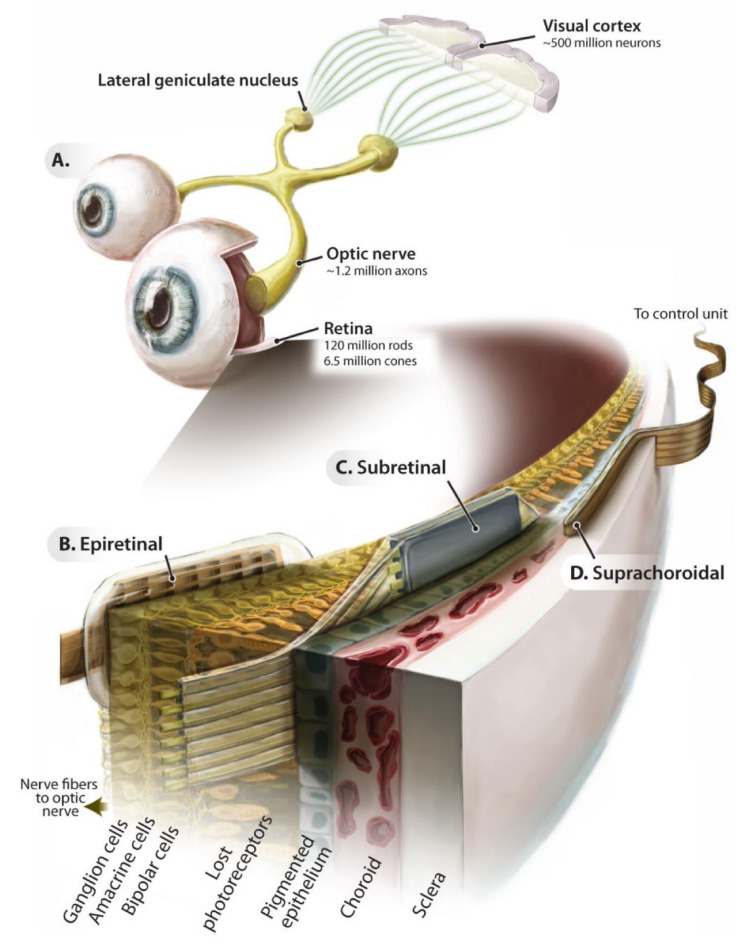
Introduction to retinal electronic prostheses. Schematic of the ascending visual pathway (**A**) and implantation locations of the epiretinal (**B**), subretinal (**C**), and suprachoroidal (**D**) implant prostheses. Adapted with permission from Eberhart Zrenner [40].

In recent years, the development of electronic retinal prosthesis has begun to draw attention. Many research teams are committed to developing retinal prosthesis, in which the chip implanted in the retina supplants the function of damaged photoreceptor cells (Figure 2) [20,21,22,23,24,25]. This is especially applicable in patients with blindness due to photoreceptor necrosis, where electrodes can be implanted in the retina to effectively stimulate bipolar or ganglion cells and subsequently enhance visual signal transmission. A retinal prosthesis chip is comprised of three structural units: a photodiode array, a photosensor with current amplifiers, and a biphasic circuit [38]. The power is mainly supplied by photovoltaic batteries, which convert incident light into electrical energy. Photoelectric conversion elements are used in lieu of damaged photoreceptor cells, transmitting electrical signals through the circuit to electrodes, such that the electrical signals stimulate cells, thereby reconstructing the visual transmission channel [39].

**Figure 3:**
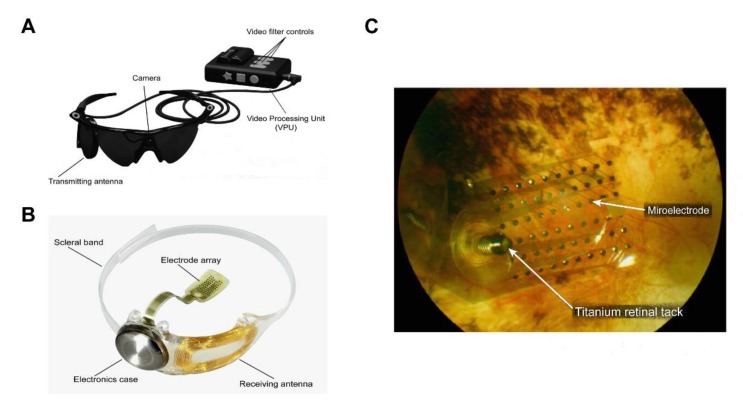
The Argus^®^ II retinal prosthesis system consists of implanted and external components. (**A**) The external components include glasses, a video processing unit (VPU), and a cable that are responsible for image capture, processing, and transmitting stimulation commands to the implant. Reprinted with permission from da Cruz, Lyndon, et al. [43]. (**B**) The implanted components include a receiver coil, electronics case, and an electrode array that are responsible for activating retinal cells. Adapted with permission from da Cruz, Lyndon, et al. [43]. (**C**) Fundus photo examination demonstrates that the microelectrode array is attached to the retina surface. Adapted with permission from Edward et al. [46].

For example, the Argus II retinal prosthesis system consists of three parts (Figure 3): (1) electronic components for optical sensing; (2) glasses with transmission function and a portable image processing device; and (3) an implantable chip with an electrode array (photodiode) [41]. The patient can capture the external image in the field of view, with the camera on the glasses, and transmit it to the image-processing device, where the image data are then converted into electronic signals and wirelessly transmitted to the electrode array chip implanted in the retina of the patient. Light energy is converted by the photodiodes on the chip into electrical pulse signals that depolarize RGCs and are finally transmitted through the optic nerve to the brain, to produce a visual image [42,43]. In particular, the retinal prosthesis chip of the first-generation Argus I system [44,45] has a 16-electrode array, which is equivalent to 16 pixels with low resolution. As a result, patients implanted with the Argus I retinal prosthesis system can only see the contour of an object and discern its directions of movement. The current second-generation Argus II system has a 60-electrode array, allowing the patients to recognize faces and read letters from the alphabet.

## 3. Challenges in Implantable Bio-Electronic Chips

Recent technological advancements in semiconductor manufacturing have made it possible to implant an electronic chip device that mimics cellular functions into the human body. This approach could solve many difficult problems in the treatment of various diseases, including treatment with surgical operations [47]. However, most of the metals used in these electrodes still require improvement in their safety, stability, and biocompatibility [48]. Therefore, only precious metals, such as platinum, gold, and silver, can be used for electrode fabrication [49,50]. However, these precious metals deposited on the electrode surface are dramatically different from the surrounding tissues, with respect to elastic mechanical properties (Young’s modulus is generally higher than 1 GPa), leading to friction and shear between the tissues and the electrode. This, in turn, significantly increases the chances of chronic inflammatory response at the site of implantation [51,52].

**Figure 4:**
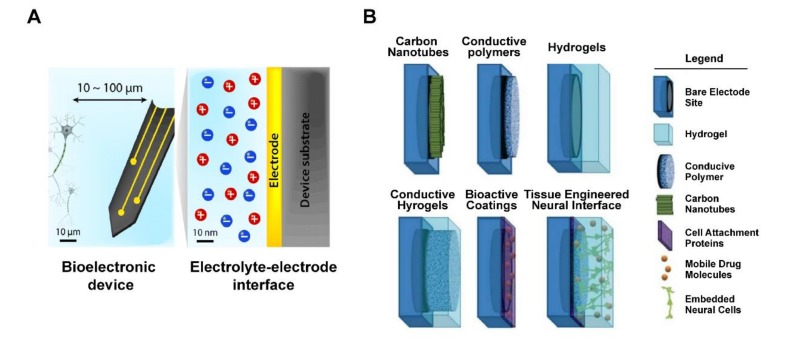
Schematic of tissue–electrode interfaces. (**A**) The electrodes of bioelectronic devices are usually implanted within 100 μm of the target tissue, and exchange of electronic signals occurs at a nanoscale electrolyte–electrode interface. Adapted with permission from Hyunwoo et al. [55]. (**B**) Application of various non-metallic material coating technologies as electrode interface, includes bare electrode site, hydrogel, conductive polymer, carbon nanotubes, cell attachment proteins, mobile drug molecules, and embedded neural cells. Adapted with permission from Aregueta-Robles et al. [57].

However, some studies have proposed modifications of the electrode surface in electronic chips, to reduce differences in mechanical properties at the interface between the metal electrode and the surrounding tissue, such that tissue damage is minimized when the device is in motion (Figure 4) [53,54]. The coating material is likely to be electrically conductive polymers, carbon nanotubes, and hydrogel membranes, all of which may provide a softer interface (Young’s modulus is generally smaller than 1 MPa) on the electrode surface than metal materials [55], thereby closer to matching biological tissue (Young’s modulus is generally between 0.1 and 10 kPa). In addition, each microchip electrode is subject to a maximum charge injection limit, which ensures that the voltage generated by the electrode is within a safe range. To improve charge-transfer efficiency and reduce constraints on charge storage, surface coating or encapsulation may be applied to the electrode materials, resulting in increased electrode surface area for charge transfer [56,57]. In addition, electrical stimulation between the tissues and the electrodes may be carried out in a safe manner, to avoid adverse chemical reactions on the electrode surface that leads to cell damage [58]. On the other hand, with the increasing demand for chip miniaturization to provide higher resolution, implants based on electrical properties of traditional electrode materials fail to overcome current problems. For example, a retinal prosthesis chip requires a charge density of 158–237 μC/cm^2^ to induce vision [59], but the charge injection-limit range of platinum is about 20–150 μC/cm^2^ [60]. This clearly indicates that the sole use of platinum is insufficient to drive the operation of retinal prosthesis. More importantly, it is not only necessary to use surface-modifying materials for improved electrical properties of the electrode surface, but it is also desirable to develop electrodes that could maintain long-term cell viability and support tissue regeneration. In this context, many studies have recently proposed the concept of incorporating living cells onto electrode surfaces [61,62,63].

## 4. Coating Materials for the Biochip Interface

In human tissues, the extracellular matrix (ECM), mainly composed of collagen, elastin, and fibronectin, provides a favorable environment for the growth, repair, support, and connection of tissues [64]. In order to enhance tissue–chip integration, some studies have used conductive hydrogels that form a soft interface on the electrode surface that provides a medium for cellular attachment [65,66,67,68,69]. This modification, in addition to facilitating cellular attachment, overcomes electrode limitations in mechanical properties, charge transfer, and charge storage. Moreover, the hydrogel networks led to an increase in the attachment percentage of astrocytes and stabilized the axonal differentiation of PC-12 cells, which is a neuronal differentiation and neurosecretion model cell line derived from a pheochromocytoma of the rat adrenal medulla, thereby regulating the growth factor and inducing gradual release of drugs [70,71,72]. In another study, a three-dimensional hydrogel structure was established to serve as a bridge linking nerve tissues, promoting the possibility of neurons growing vertically toward the tissues [73]. Other research efforts were made to combine ECM with graphene derivatives. Due to its polyfunctional groups and high hydrophilicity, graphene oxide (GO) forms a stable hydrogel structure, which promotes mechanical strength and swelling properties of proteins through cross-linked bonding (i.e., van der Waals forces, electrostatic attraction, and hydrogen bonding), while sustaining a microenvironment suitable for cell and tissue growth [74,75]. These studies indicated that GO-based composites provide a promising new material for assembling suitable cellular microenvironments and should be explored in biochip-interface applications [76,77].

## 5. Graphene Oxide (GO)

Graphene is a two-dimensional nanomaterial sheet composed of a hexagonal lattice structure formed by the stacking of single carbon atoms. Carbon atoms are bonded to each other through sp^2^ hybridization. Due to its unique physical and chemical properties, including excellent mechanical strength, electron mobility, thermal conductivity, specific surface area, and light transmittance [78,79], graphene has drawn considerable attention and has been applied to various biomedical fields, such as electronic components and semiconductors [80,81], biosensors [82,83], nanocomposites [84], and drug-delivery vehicles [85].

Among graphene derivatives, graphene oxide (GO) is the oxidation product of graphene, with a structural composition similar to graphene, except for the presence of a number of surface oxygen functional groups, such as hydroxyl (C-OH), epoxy (COC), carboxyl (COOH), and carbonyl (C=O) groups, making GO mixture of sp^2^ and sp^3^ bonding [86]. The region with sp^2^ bonding represents a stable arrangement of carbon atoms, while the region with sp^3^ bonding represents the distribution of oxygen groups at sites of carbon structural defects. In particular, the abovementioned oxygen functional groups render the surface of GO nanomaterials hydrophilic and promote their bonding with other materials, including microscopic objects, such as quantum dots, antibodies, nanoparticles, and peptides. In addition, because of its good biological compatibility and richness in poly-functional groups for chemical modification, GO has been widely used in cell biology experiments, and has found applications in drug delivery, tissue engineering, and bio-imaging. This has led to GO being considered as a multifunctional nanomaterial of great development potential in the biomedical field [87,88,89,90].

## 6. Advantages of the GO Interface

GO has good physical and chemical properties. At present, many biosensor studies have explored the use of single-layered GO nanosheet as a chemical modification platform, based on the excellent hydrophilicity, electrical conductivity, specific surface area, and chemical stability of this material [91]. For example, the high specific surface area of GO (736.6 m^2^/g) is superior to conventional 2D materials (e.g., aluminum oxide, titanium, and silica) [92], making the GO surface a biologically active interface where various surface oxygen functional groups can readily bind to biomolecules [93,94]. Electrochemical measurements have been carried out to determine the properties of GO-bonded substances, including dopamine, DNA, NADH, which is an important cofactor of metabolism in all living cells, heavy metal ions, and enzymes [94]. These studies have reported a relationship between the current of redox reaction and the concentration of the bonded substances. Studies have demonstrated the use of GO sheet as a glucose biosensor, by inducing covalent bonding of GO-activated carboxyl groups to the amino groups of glucose oxidase, with excellent detection sensitivity and reproducibility [95,96]. Moreover, our previous studies have shown that retinal pigment epithelial (RPE) cells cultured for 72 h on the surface of GO electrode exhibited good adhesion and differentiation properties. This suggested that the GO interface exhibits good tissue compatibility and low levels of cytotoxicity, which can be potentially ideal materials as long-term bioelectrodes [97].

## 7. GO Biocompatibility

Currently, many research teams have begun exploring the interaction between GO as a surface material and organisms by performing in vitro and in vivo biocompatibility and toxicity assessments [98,99,100]. The most common method for preparing GO is based on the strategy proposed by Hummers et al., in 1958 [101]. The average lateral dimension of GO synthesized using this method will vary with chemical conditions of graphene treatment, such as reaction time, temperature, concentration, and centrifugal speed, which results in changes in surface morphology and thickness of the GO nanosheet. Although the GO thickness is 1.1 nm, the average lateral sizes of GO are 205.8, 146.8, and 33.8 nm, respectively, which indirectly affect the survival rate of epidermal cancer cells (HeLa) [102]. When the GO concentration is kept the same at 20 μg/mL, GO with a size of 205.8 nm has high cytotoxicity, while 146.8 nm GO has lower cytotoxicity than 33.78 nm GO, implying that small-sized GO may cause less damage to cell membranes [102]. On the other hand, studies have shown that single-layer and multilayered GO exhibited different colloidal behaviors in cell culture medium [103]. The results show that the surface area of single-layer GO can interact with fetal bovine serum (FBS) protein at a much higher rate than multilayered GO. This means that different layers of GO have significant differences in surface hydrophobicity and dispersion, which will seriously affect the biological activity of corona. In addition, some relevant studies have compared the effects of in vitro and in vivo concentration of GO on cell biocompatibility. These showed that, for human diploid fibroblast (HDF) cells cultured with different doses (10, 20, 50, and 100 μg/mL) of GO, GO exhibits low cytotoxicity, with > 80% cell survival rate when GO concentration is below 20 μg/mL [104]. When the GO concentration is above 50 μg/mL, noticeable cell apoptosis occurs, which may be attributed to loss of function of lysosome, mitochondria, and endoplasmic reticulum. In another study [104], mice were subjected to intravenous injection of GO suspension in the tail and observed at 1, 7, and 30 days of culture. Lung tissues of these mice collected for hematoxylin–eosin staining and observation revealed that a low dose (0.1 mg) of GO did not have significant toxicity to the mice. However, a high dose (0.4 mg) led to accumulation of GO in the lungs and liver, and the accumulated GO could not be removed from the body through physiological processes, resulting in a 30 days survival rate below 60% in these mice. In short, follow-up in vitro (< 20 μg/mL) and in vivo (< 0.1 mg) experiments with low concentration of GO have proven that low-concentration GO has good biocompatibility and can be used as a biointerface for tissue engineering.

## 8. GO Potential for the Retina

In recent years, research teams have begun to test the possibility of using GO as a drug-delivery vehicle, conducting in vitro and in vivo biocompatibility assessments of GO to human retinal pigment epithelium (RPE) cells. In the in vitro experiments, cell survival rates gradually decreased from the initial ~85% to 60%, with increasing concentrations (5–100 μg/mL) of GO added to cultured RPE cells [105]. In addition, in vivo experiments revealed that vitreous injection of three low levels of GO (0.1, 0.2, and 0.3 mg) in rabbits, followed by 49 days of culture, did not cause obvious ocular structural defects or any inflammatory reaction, as confirmed by the examination of eyeball appearance and intraocular pressure (IOP), as well as electroretinography (ERG) [105]. IOP and visual acuity monitored for seven weeks after the injection were not significantly different from those in the control group, indicating that GO had no effect on IOP. ERG studies further confirmed that there was no significant decrease in visual acuity, with the amplitude varying by less than 30%, which is considered negligible. Inspection of histological sections revealed that GO was not deposited in the retina, thus demonstrating that low-concentration GO had good biocompatibility in the eye. In the meantime, some studies explored the use of GO as a fluorescent nanosensor to detect lysine content in RPE cells [106]. Alizarin red was covalently attached to GO and co-cultured with cells for 1 h, after which the system was illuminated with light, to induce electron transfer. This electron transfer released lysine, which reacted with alizarin red, to generate red fluorescence, enabling instant monitoring of the electron transfer reaction in RPE cells with fluorescence microscopy imaging. In short, GO has excellent biomedical-application potential for a wide range of processes, ranging from retinal cell imaging to drug delivery.

## 9. Applications of GO Micropatterns

At present, many research teams use ECM to prepare micropatterns to mimic in vivo regulation of interactions between cells and proteins, including cell migration and differentiation [107,108,109]. However, the interaction between cells and ECM is subject to unstable physical and chemical factors, such as low mechanical strength with high degradation susceptibility, preventing long-term culture; different protein concentration gradients; and oxidative protein denaturation. All of these unstable factors may affect the physiological functions of cells [110]. Therefore, some research teams have proposed using high-biocompatibility polymers or nanomaterials, to prepare micro patterns. These polymers and nanomaterials have stable physical properties, including high functional groups distribution, mechanical strength, and surface roughness, which is benefit to cellular morphology, proliferation, and differentiation behavior of cells [111,112,113,114]. To date, there have been many methods to prepare geometric micropatterns of various materials. In these methods, biological materials are used to control cellular arrangement and positioning on the surface of various substrates, including gold [115], glass [116], silicon wafers [117], and polymers [118], with the micropatterns widely applicable to tissue engineering, cellular drug screening, cell microarrays, and biosensors. It is possible to prepare patterns mimicking the cellular microenvironment by using various high-precision processing techniques, such as soft lithography [119], electron beam lithography [120], microcontact printing [121], self-assembled monolayer [122], and inkjet printing [123].

**Figure 5:**
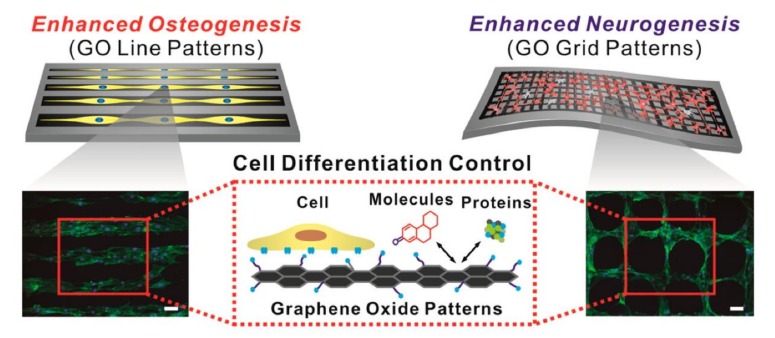
Effects of GO micropatterns on stem-cell differentiation. Enhancement of osteogenic and neuronal differentiation, using microcontact-printed GO substrate with line and grid patterns. Fluorescence image of stem cells stained with F-actin. Scale bar = 100 μm. Reprinted with permission from Kim et al. [125].

In recent years, the application of GO in biochips has seen a rising trend of surface micropatterning. There are a number of reports of using GO to prepare micropatterns to promote cellular processes, such as directional migration of cells [124], axonal extension and arrangement of neural stem cells (NSCs), and differentiation of NSCs into neurons [112], as well as regulation of the differentiation direction of human-adipose-derived mesenchymal stem cells (hADMSCs) [125,126]. The attachment of hADMSCs to a linear GO pattern has been shown to enhance osteogenic differentiation, and a grid-like GO pattern facilitates the differentiation of hADMSCs into neurons, due to the ability of the grid patterns to mimic interconnected/elongated neuronal networks. (Figure 5). On the other hand, many previous studies [94,127,128] have shown that graphene has the ability of non-covalent bonding (π-π stacking interactions), while GO has the ability to make electrostatic interactions and hydrogen bonds. The different degree of oxygen content can be mediated to change the binding capacity of growth agents (dexamethasone, β-glycerophosphate, insulin, and ascorbic acid) during the differentiation of stem cells. In addition, Yang et al. [129] used photolithography to prepare GO in a patterned groove substrate, exploring the effect of roughness and surface morphology of the GO microstructure in promoting differentiation of NSCs; the result showed that NSCs will grow along the GO attached in the groove. Immunofluorescence staining revealed that, when the groove width in the pattern is below 5 μm, NSCs exhibit a good arrangement of cytoskeletal F-actin, aggregation of β1 integrin, and expression of neuronal markers (neuron-specific beta-III tubulin (Tuj-1) and microtubule-associated protein 2 (MAP2)). This observation indicates that the unique physicochemical properties of GO combined with the surface topography of microstructured patterns can stimulate the interaction between stem cells and biomaterials and enhance the ability to induce differentiation of neurons. Therefore, GO micropatterns may be applied to nerve-tissue engineering and stem-cell therapy.

## 10. Application of GO Micropatterns to the Retina

**Figure 6:**
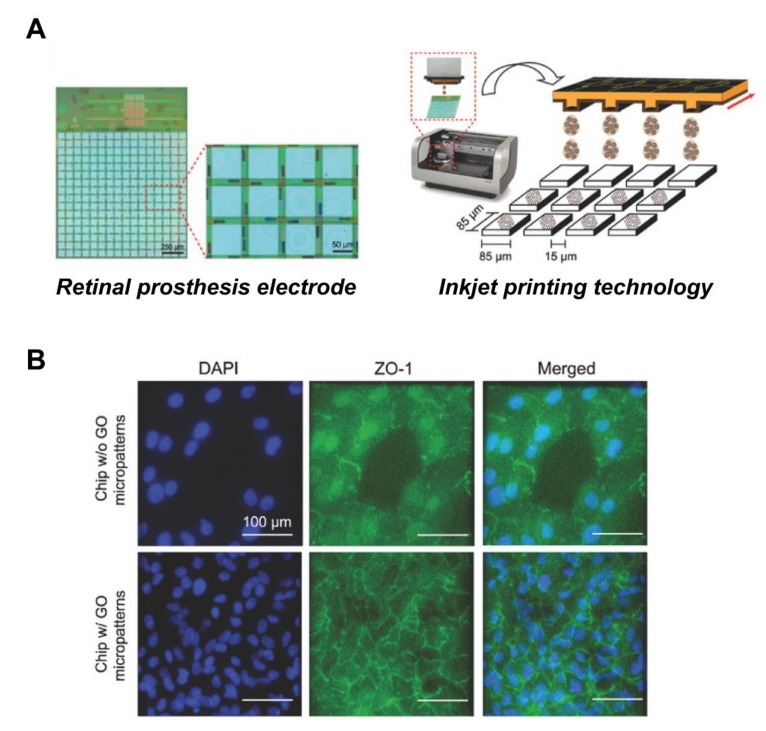
Improved biocompatibility of RPE cells with retinal prosthesis electrode attachment through GO patterns. (**A**) Schematic of the GO micropatterns generated on the electrodes by inkjet-printing technology. (**B**) Fluorescence image of cells stained with tight junction protein marker, ZO-1. The GO-micropatterned interface showed enhanced cell adhesion and barrier function on the retinal prosthesis electrode substrate. Scale bar = 100 μm. Adapted with permission from Yang et al. [97].

Bendali et al. used UV-lithography to prepare a linear graphene pattern, with a width of about 10 μm, and cultured RGCs on this patterned material, finding that the axons grew along the linear pattern [130]. Moreover, Yan et al. [131] used an electrospinning method to prepare a graphene nanofiber composite, finding that RGCs were arranged along the direction of nanofibers. Through electrical stimulation experiments, they further evaluated the possibility that electrical stimulation would promote axon regeneration and cellular morphological changes, confirming that, under electrical impulse stimulation (±700 mV/cm), the axon length of RGCs increased from 50 to 107 μm, and the survival rate of RGCs was more than 80%. This shows that appropriate electrical stimulation of retinal cells is beneficial for axon growth, and that it is possible to combine retinal prosthesis with GO, to provide follow-up electrical stimulation. Recently, our group designed GO micropatterns with sizes compatible to the electrode matrix of retinal prosthesis chip, and successfully employed inkjet-printing technology, to print GO micropatterns with diameters of 30 and 80 μm on retinal prosthesis [97]. These GO micropatterns were served as a nanoscale biointerface for the attachment of cells, so as to achieve cell patterning (Figure 6). The retina cells were successfully attached in a specific area of the electronic chip, using the above fabricated nano-biointerface, indicative of a prototype for retinal prosthesis. In the future, retinal prosthesis chips will need to focus more on exploring whether electrical stimulation will promote intercellular connectivity between cells and biological electrodes, thereby repairing damaged retinal structure and enhancing the clinical application value of retinal prosthesis chips.

## 11. Outlook

**Figure 7:**
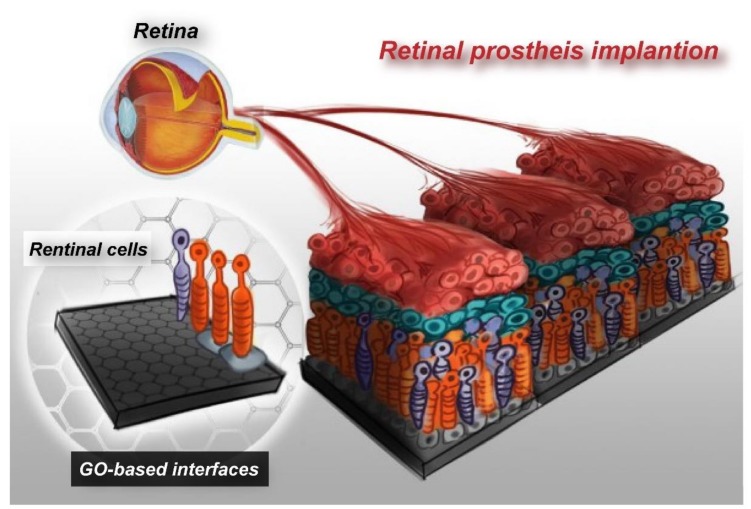
Concept of the advanced electronic retinal prosthesis. Establishing GO-based electrode interfaces with embedded retinal cells, which has the potential to improve the efficiency of retinal prosthesis tissue regeneration and repair functions.

Recently, due to the rapid technological advancement in semiconductor manufacturing, it has become possible to implant an electronic chip device that mimics cellular functions into the human body, as a tentative treatment strategy for retinopathies. However, most of the electrode materials require improvement in their safety, stability, and biocompatibility. GO, as a nanomaterial with high biocompatibility and superior physiochemical properties, has great potential for application to the electrodes of retinal prosthesis. Provided that retinal prosthesis is integrated with GO nanopatterns, it will be possible to attach retinal cells on a specific chip electrode and, thereby, upgrade retinal prosthesis into bioelectronic retinal prosthesis. The effects of electrode current on cellular attachment, proliferation, and survival rate, and even the effects on the connectivity between cells attached to the chips, remain to be explored. Micropatterned GO interface will facilitate cellular adhesion on retinal prosthesis, which, in turn, will help improve tissue regeneration and repair functions, enhance safety associated with retinal prosthesis chip implantation, and overcome immunological rejection (Figure 7). This represents a big cross-disciplinary breakthrough in chip design. This innovative nano-interface technology is expected to enhance the clinical application value of retinal prosthesis in future.
